# Breast tumor copy number aberration phenotypes and genomic instability

**DOI:** 10.1186/1471-2407-6-96

**Published:** 2006-04-18

**Authors:** Jane Fridlyand, Antoine M Snijders, Bauke Ylstra, Hua Li, Adam Olshen, Richard Segraves, Shanaz Dairkee, Taku Tokuyasu, Britt Marie Ljung, Ajay N Jain, Jane McLennan, John Ziegler, Koei Chin, Sandy Devries, Heidi Feiler, Joe W Gray, Frederic Waldman, Daniel Pinkel, Donna G Albertson

**Affiliations:** 1 Department of Epidemiology and Biostatistics, University of California San Francisco, San Francisco, San Francisco, CA 94143, USA; 2 University of California San Francisco Comprehensive Cancer Center, San Francisco, CA 94143, USA; 3 Cancer Research Institute, University of California San Francisco, San Francisco, CA 94143-0808, USA; 4 Micro Array Core Facility, VUMC University Medical Center, 1081BT Amsterdam, The Netherlands; 5 Department of Epidemiology and Biostatistics, Memorial Sloan-Kettering Cancer Center, New York, New York 20021, USA; 6 Department of Laboratory Medicine, University of California San Francisco, San Francisco, CA 94143-0808, USA; 7 Geraldine Brush Cancer Research Institute, California Pacific Medical Center, San Francisco, California 94115, USA; 8 Department of Pathology, University of California San Francisco, San Francisco, California, USA; 9 Division of Life Sciences, Lawrence Berkeley National Laboratory, Berkeley, CA, USA

## Abstract

**Background:**

Genomic DNA copy number aberrations are frequent in solid tumors, although the underlying causes of chromosomal instability in tumors remain obscure. Genes likely to have genomic instability phenotypes when mutated (e.g. those involved in mitosis, replication, repair, and telomeres) are rarely mutated in chromosomally unstable sporadic tumors, even though such mutations are associated with some heritable cancer prone syndromes.

**Methods:**

We applied array comparative genomic hybridization (CGH) to the analysis of breast tumors. The variation in the levels of genomic instability amongst tumors prompted us to investigate whether alterations in processes/genes involved in maintenance and/or manipulation of the genome were associated with particular types of genomic instability.

**Results:**

We discriminated three breast tumor subtypes based on genomic DNA copy number alterations. The subtypes varied with respect to level of genomic instability. We find that shorter telomeres and altered telomere related gene expression are associated with amplification, implicating telomere attrition as a promoter of this type of aberration in breast cancer. On the other hand, the numbers of chromosomal alterations, particularly low level changes, are associated with altered expression of genes in other functional classes (mitosis, cell cycle, DNA replication and repair). Further, although loss of function instability phenotypes have been demonstrated for many of the genes in model systems, we observed enhanced expression of most genes in tumors, indicating that over expression, rather than deficiency underlies instability.

**Conclusion:**

Many of the genes associated with higher frequency of copy number aberrations are direct targets of E2F, supporting the hypothesis that deregulation of the Rb pathway is a major contributor to chromosomal instability in breast tumors. These observations are consistent with failure to find mutations in sporadic tumors in genes that have roles in maintenance or manipulation of the genome.

## Background

Genomic DNA copy number aberrations are frequent in solid tumors [1]. The wide range in the number and types of chromosome level alterations are likely to reflect the different solutions taken by individual tumors to escape normal protective mechanisms. Thus, the spectrum of alterations is likely to reflect a composite of selection and particular failures in genome surveillance mechanism(s). The interplay between selection and genetic instability in shaping tumor genomes is currently most clearly established in tumors with defects in mismatch repair. These tumors have a high frequency of nucleotide sequence level aberrations, fewer DNA copy number alterations and characteristic histological phenotype [1]. On the other hand, less is known about specific gene defects that give rise to chromosome level aberrations in tumors. Mutations in genes encoding proteins involved in mitosis and DNA damage sensing and repair mechanisms, which are associated with chromosomal level instability have been identified in cancer-prone syndromes, including *ATM*, *TP53*, *BRCA1*, *BRCA2*, *NBS1 *and *BUB1B*, however they are rarely mutated in sporadic tumors [2,3]. Similarly, searches for mutations in genes that participate in maintenance or manipulation of the genome (e.g. genes involved in DNA repair, replication, spindle checkpoints etc.) have found only a small number of mutations in tumors [3]. Nevertheless, deregulation of functions that maintain genome stability appears to occur early in tumors, as activation of the DNA damage checkpoint, possibly in response to DNA replication stress, is evident in pre-malignant lesions [4,5]. Similarly, telomere shortening is observed in pre-malignant lesions, supporting a role for telomere dysfunction early in tumor development [6]. Other proposed routes to instability include deregulation of *CCNE1 *and *AURKA *expression through loss of function of *FBXW7 *(hCdc4) [7] and more global alteration in gene expression due to deregulation of the Rb pathway [8]. The foregoing discussion suggests that failures in a number of different processes that maintain genome integrity could contribute to the wide variety of genomic alterations in solid tumors. Often these aberrations include net gain or loss of whole chromosomes (aneuploidy) or parts of chromosomes. Gene amplification, defined as a copy number increase of a restricted region of a chromosome arm may also occur. Here we investigated the numbers and types of copy number alterations in tumors and whether they were associated with differential expression of genes likely to play a role in manipulation or maintenance of the genome. These studies found three subtypes of breast tumors distinguished by copy number aberrations. Telomere dysfunction was implicated in the propensity to amplify, since shorter telomeres and differential expression of genes involved in telomere maintenance were associated with the numbers of amplicons and the presence of at least one amplicon, respectively. On the other hand, the number of lower magnitude gains and losses of chromosomal segments was associated with differential expression of genes involved in processes maintaining or manipulating the genome. These genes are significantly enriched for the known targets of E2F. Furthermore, we observed enhanced expression of most E2F target genes, indicating that over expression rather than deficiency was associated with genetic instability. These observations support the hypothesis that deregulation of the Rb/E2F pathway is a major contributor to chromosomal instability in breast tumors.

## Methods

### Specimens

Frozen tumor tissue was obtained from the University of California San Francisco Comprehensive Cancer Center Breast Oncology Program Tissue Bank. All specimens were collected under approved protocols from UCSF with patient consent. Patient characteristics are provided in Supplementary Table 1 (Additional file 1). Expression and copy number data from a second set of ductal invasive breast tumors were used and patient characteristics are given in Chin *et al. *(submitted). The patient groups in both sets were similar in terms of their genomic and pathological characterization.

### Extraction of nucleic acids

Nucleic acids were extracted from tumor blocks as described previously [9,10]. Briefly, blocks were trimmed with a razor blade to remove normal tissue and cryosections were obtained from either side of the block to ascertain that tumor cells comprised greater than 70% of the specimen. DNA was extracted using the QUIamp tissue kits (29304, Qiagen).

### *TP53 *sequencing

Exons 5–8 of *TP53 *were amplified from genomic DNA and cycle sequencing was carried out as described previously [11].

### Array CGH and data processing

Array CGH, imaging and data analysis were carried out as described previously using arrays of 2464 genomic clones (BAC or P1) each printed in triplicate (HumArray1.14 and HumArray2.0) [11,12]. Data processing is described in detail in the Supplementary Methods (Additional file 2) and the array data are available in Supplementary Table 2 (Additional file 3).

### Telomere length assessment

The mean TRF length was measured using the TeloTAGGG telomere length assay kit (Roche Applied Science). Briefly, 1 μg genomic DNA was digested with *Hin*f I and *Rsa *I restriction enzymes and electrophoretically resolved on 0.8% agarose/1X TAE. The gels were blotted to a nylon membrane (Positive charged, Roche) and fixed by UV-crosslinking. After hybridization with digoxigenin labeled telomere specific probe, the signals were visualized with an alkaline phosphatase – CDP-Star chemiluminescent system. The filters were exposed to X-ray film and the mean TRF length was calculated using Quantity One software.

### Statistical methods

A detailed description of the methods used for all aspects of the data analysis is provided in the Supplementary Methods (Additional file 2).

## Results

### Genomic analysis of breast tumors

**Figure 1:**
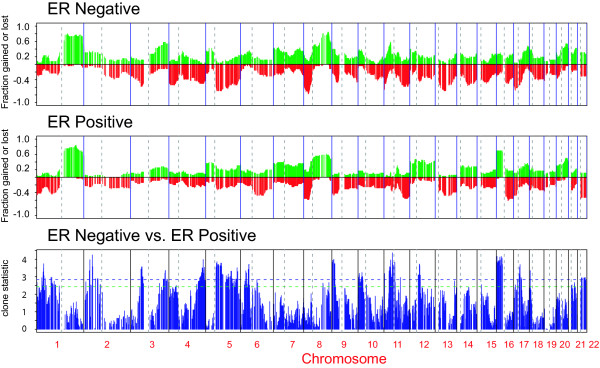
**Frequency plot of copy number alterations in ER positive and negative tumors**. The top two panels show the frequency of gains, indicated by the green bars ranging from 0 to 1, and losses, indicated by the red bars ranging from 0 to -1, in 62 sporadic breast tumors for each clone. The bottom panel displays the magnitude of the t-statistic for each clone computed based on the smoothed data as described in the Methods. The horizontal dotted lines indicate the statistic cut-off corresponding to the FDR-adjusted p-value of 0.05 (blue) and 0.1 (green).

**Figure 2:**
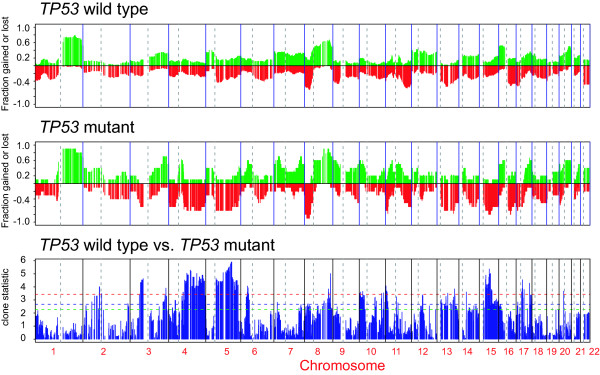
**Analysis of TP53 mutation in breast tumors**. Frequency plot of copy number changes in *TP53 *mutant and wild type tumors. The top two panels show the frequency of gains, indicated by the green bars ranging from 0 to 1, and losses, indicated by the red bars ranging from 0 to -1, in 62 sporadic breast tumors for each clone. The bottom panel displays the magnitude of the t-statistic for each clone computed based on the smoothed data as described in the Methods. The horizontal dotted lines indicate the statistic cut-off corresponding to the FDR-adjusted p-value of 0.01 (red), 0.05 (blue) and 0.1 (green). Thus, copy number alterations occurring more frequently in *TP53 *mutant tumors included losses of regions on 3p, 4q, 5q, 15q, 17q and gain of a small region on distal 8q.

Application of array CGH to the analysis of copy number aberrations in 62 sporadic ductal invasive breast tumors and five *BRCA1 *mutant tumors revealed a number of frequent low level gains and losses (Supplementary Tables 1 and 2, Additional files 1 and 3) and 12 regions of recurrent amplification (Table 1). We observed significant differences in the spectrum of aberrations with respect to estrogen receptor (ER) status (Figure 1) consistent with other published reports [13]. We also found that tumors with mutations in exons 5–8 of *TP53 *showed a higher frequency of alterations, as well as significant differences in the frequency of alteration of certain regions of the genome compared to tumors without mutations as indicated by the t-statistic for each clone (Figure 2). Moreover, we found a highly significant concordance between the test statistic for association of the particular chromosomal regions with *TP53 *mutation status in our data and an independent set of more than 100 primary breast tumors (Fedele *et al*., personal communication) (Pearson correlation of 0.53 corresponding to the p-value << 0.001).

**Table 1:** Recurrent amplicons in breast tumors with examples of some candidate oncogenes

**Tumors**	**Chr.**	**Proximal flanking clone**	**Start (bp)**	**Distal flanking clone**	**End (bp)**	**Size (Mb)**	**Candidate Genes**
S0257; S1508	1	RP11-235B24	50120611	CTD-2010C4	64641284	14.5	*JUN*
S0021; S0065; S0127; S0132; S1534; S1539	8	RP11-210F15	36452678	RP11-262I23	39744917	3.3	*BAG1; FGFR1; TACC1*
S0013; S0257; S1598	8	RP11-128G18	127638988	RP11-227F7	131013284	3.4	*MYC; WISP1*
S0132; S0394; S1524	11	CTD-2115C17	32421376	RP11-18B9	40252688	7.8	*TRAF6*
S0050; S0065; S0081; S0132; S0252; S0303; S1534; S1539; S1598	11	CTD-2080I19	68483419	RP11-98G24	76964746	8.5	*CCND1; FGF4; EMS1; PAK1*
S0184; S1508	12	12pter	0	RP11-272L6	5221281	5.2	*CCND2; FGF6; DYRK4*
S0051; S0052; S0122; S1522	12	RP11-18B8	63882712	RP11-92P22	74052886	10.2	*MDM2; DYRK2; YEATS4; HELB;*
S0021; S0043; S0052; S0059; S0257; S0394; S1511; S1522; S1526; S1539	17	RP11-58O8	34147697	RP11-87N6	38680670	4.5	*STARD3; ERBB2; GRB7; TOP2A; MMP28*
S0043; S0104; S0394	17	RP11-110H20	47546526	RP11-481C4	49705574	2.2	*IMP-1; ITGA3*
S0001; S0021; S0043; S0104; S1508; S1511	17	RP5-1073F15	58499308	CTB-244K7	64939230	6.4	*BRIP1; GH1; GH2; MAP3K3*
S0127; S0257; S0269	18	RP11-7E5	10817140	RP11-10G8	17272427	6.5	*PTPN2*
S0021; S0043; S0050; S0051; S0055; S0059; S0122; S1522; S1545; S1598	20	RP11-169A6	44424925	RMC20P179	62702798	18.3	*CYP24; ZNF217; STK6*

**Figure 3:**
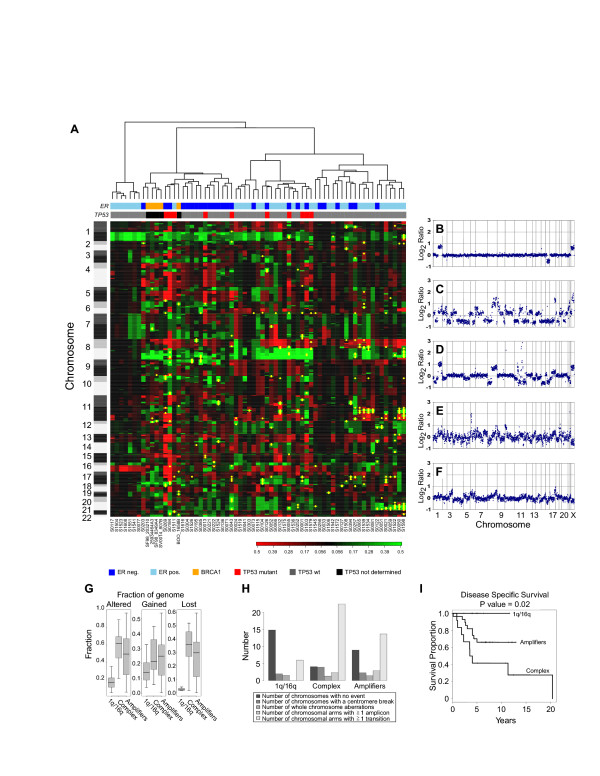
**Genomic analysis of breast tumors reveals three subtypes**. **A**. Hierarchical clustering of 62 ductal invasive breast tumors and five *BRCA1 *mutant tumors based on their genome-wide DNA copy number profiles. Individual clones are represented as rows, ordered by chromosome and genome position according to the July 2003 freeze of the human genome. Clones on the p-arm and q-arm of chromosomes are indicated in shades of dark gray (odd numbered chromosomes) or light gray (even numbered chromosomes). Acrocentric chromosomes are shown in dark or light gray. Columns represent individual tumor samples. The estrogen receptor status of the tumors is shown in shades of blue (dark blue = ER negative, light blue = ER positive), *BRCA1 *mutant tumors are indicated in orange, and *TP53 *mutation status is indicated with a maroon box for *TP53 *mutant tumors, a gray box for tumors with no detected mutation and a black box if the *TP53 *status is unknown. Copy number losses are indicated in red, gains in green and amplifications as yellow dots. Three main clusters are evident. **B-F**. Genome-wide copy number aberrations profiles of sporadic and hereditary (*BRCA1*) breast tumors are plotted as the normalized log_2_ratio for each clone sorted by chromosome and ordered according to genome position from the p-arm to the q-arm. Normalized copy number ratios of genomic DNA are shown for a tumor from the 1q/16q cluster with few copy number changes including gain of 1q and loss of 16q (B), a tumor from the ER negative, complex cluster showing many low level chromosome changes and few amplifications (C), a tumor from the amplifier cluster with low level gains and losses and amplifications (D) and tumors from patients with mutations in *BRCA1 *(E and F). **G**. Numbers and types of copy number aberrations in breast tumor subtypes. The mean numbers of whole chromosome copy number changes, copy number transitions and amplifications were determined for the tumors within each subtype. **H**. Numbers and types of copy number aberrations in breast tumor subtypes. The mean numbers of chromosomes showing no copy number change, whole chromosome copy number changes, copy number transitions, copy number transitions at centromeres and amplifications were determined for the tumors within each subtype. **I**. Association with disease-specific survival. Significance of the log-rank test was used to assess the association between a genomic subclass and survival phenotypes. The significance was declared at p < 0.05. Patients with complex tumors experienced significantly worse outcome compared to the other groups.

**Figure 4:**
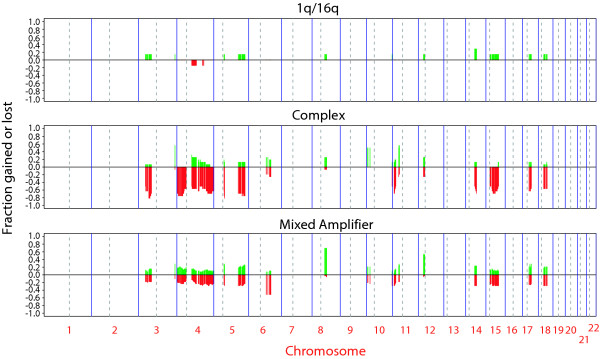
**Copy number changes more frequently associated with one subtype**. Frequency for each clone of gains and losses, which were uniquely present in more than 50% of samples of one subtype and in less than 30% of samples in other subtypes. Gains are indicated by green bars, ranging from 0 to 1, and losses, by the red bars ranging from 0 to -1 for each clone.

Hierarchical clustering of tumors according to their genome-wide DNA copy number profiles revealed three main branches. Tumors within each of the branches also differed in the number of copy number changes that were present, as well as the frequency of particular aberrations (Figures 3 and 4). The same three clusters were observed in an independent set of breast tumors (Chin *et al*., submitted), thus confirming our initial observation. The groups in both sets agreed in terms of their genomic and pathological characterization.

At the low end of chromosomal level instability are ER positive tumors (n = 7 tumors, Figure 1A, left branch), designated 1q/16q, as their genomes showed very few copy number changes other than gain of 1q and loss of 16q (Figure 3A, B, G and 3H, Figure 4). Tumors in this group were exclusively of moderately or well differentiated grade, stage II, and did not recur. These tumors had very high within group similarity with average pairwise Pearson correlation of 0.76.

At the other extreme of genome instability are sporadic tumors (n = 16, Figure 3A, middle branch) in which the mean fraction of the genome at altered copy number is greater than 0.6 due to the presence of many low level copy number aberrations (Figure 3A, C, G and 3H). Copy number losses involving chromosomes 3p, 4, 5q, 11p, 14q, 15q, 17q and 18q were more prevalent in this subtype than in others (Figure 4). All but one tumor in this group are ER negative, all were of high grade and patients experienced significantly worse outcome as compared to other groups (Figure 3I). Four had mutations in exons 5–8 of *TP53*, accompanied by a copy number loss encompassing the locus (Table 2). In addition this cluster contains all of the familial *BRCA1 *mutant tumors (Figure 3E and 3F) in our dataset. Similar to *BRCA1 *mutant tumors, they show a relatively high degree of within group similarity in regions of aberration in spite of the presence of many alterations (see Methods for discussion of statistical analysis, Additional file 2). We refer to this group as "complex" in recognition of their many low level copy number alterations.

**Table 2:** *TP53 *mutations in breast tumors

**Tumor Sample**	**CGH Subtype**	***TP53 *Mutation**	**Copy Number**	**Loss of Function**^1^	**Gain of Function**^1^	**Dom. Neg.**^1^	**TS**^1^
S0013	Complex	Y205D	Loss	NA	NA	NA	NA
S0184	Complex	R175H	Loss	Yes	Yes	Yes	No
S0269	Complex	M237I	Loss	NA	NA	No	NA
S1511	Complex	C275Y	Loss	NA	NA	No	NA
S0001	Mixed amplifier	Y234C	No Loss	Yes	NA	NA	Yes
S0043	Mixed amplifier	Y163C	No Loss	Yes	Yes	No	NA
S0055	Mixed amplifier	196 Stop	Loss	NA	NA	No	NA
S0126	Mixed amplifier	intron 5/6	Loss	NA	NA	NA	NA
S1503	Mixed amplifier	C176Y	Loss	Yes	NA	Yes	NA
S1579	Mixed amplifier	G245C	Loss	Yes	NA	Yes	NA

^1^From IARC TP53 database release, R10, July 2005 [48]. Function assayed in either human or yeast. Dom. Neg., Dominant Negative. TS, Temperature sensitive.

**Figure 5:**
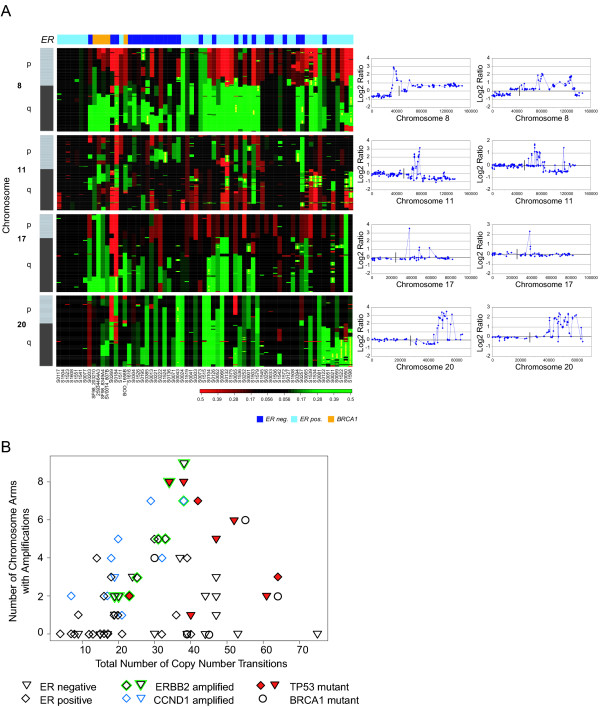
**Overview of most frequent DNA amplifications on chromosomes 8, 11, 17 and 20 in 62 breast tumors determined by genome-wide array CGH**. **A. **Heat map depiction of aberrations on chromosomes 8, 11, 17 and 20 and typical chromosome copy number profiles showing amplifications of 8p (including *FGFR1*), 11q13 (including *CCND1*), 17q (*ERBB2*) and regions on 20q (including *ZNF217*). Note that the chromosome 11 copy number profiles vary depending on whether amplification of chromosome 8 is also present. In both cases copy number losses distal to the amplicon are observed, however in the absence of chromosome 8 amplification (right), the region of loss extends distally from the amplified region, whereas, when chromosome 8 was amplified (left), the copy number loss includes regions proximal as well as distal to *CCND1*. **B. **For each tumor, the numbers of copy number transitions is compared to the number of chromosome arms with at least one amplification.

The third group (n = 39, Figure 3A, right branch) comprised of both ER positive and negative tumors is characterized by the presence of low level gains and losses and recurrent amplifications (Figure 3A, D, G and 3H). Gains involving chromosome 8q and 12p were more frequent in this group than the others (Figure 4). We refer to this group as "mixed amplifiers." The more frequently occurring amplifications in this group, which occurred predominantly in the ER positive tumors involved 8p, including *FGFR1 *(6 ER positive tumors/6 tumors with 8p amplification), 11q13, including *CCND1 *(8 ER positive tumors/9 tumors with 11q13 amplification) and regions of 20q including *ZNF217 *(6 ER positive tumors/9 tumors with 20q amplification). Within this subtype, amplification of 17q (*ERBB2*) was present in both ER positive (n = 5) and ER negative tumors (n = 2) (Figure 5A and Table 1).

### Association of copy number aberration types with alterations in processes/genes involved in maintenance and manipulation of the genome

**Figure 6:**
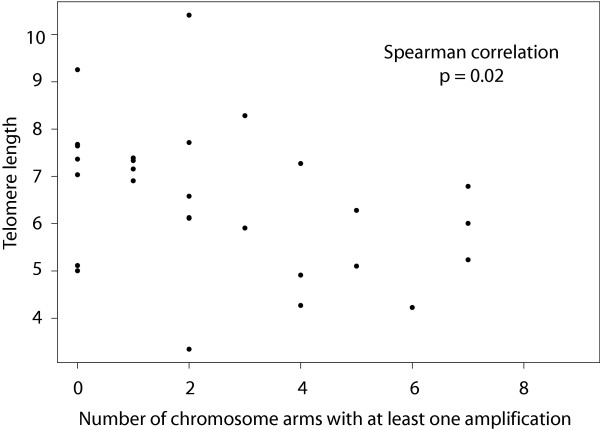
**Telomere length measurements in 28 breast tumor samples**. Plotted is the telomere length determined by Southern blotting relative to the number of chromosome arms with amplification. A significant inverse correlation is evident.

The discrimination of breast tumor subtypes based on copy number aberrations led us to investigate possible associations of copy number aberration types with alterations in processes/genes involved in maintenance of genome stability. Over expression or depletion of such genes *in vitro *results in a variety of genome instability phenotypes, including disruptions of chromosome integrity, aberrant mitoses, aberrant cell division, etc. As telomere dysfunction has been widely proposed as a source of genetic instability in tumors, we first investigated the possible association of telomere attrition with copy number aberrations. We determined average telomere length in 28 breast tumors using Southern blotting. We found an inverse correlation between telomere length and number of chromosome arms with amplification (Figure 6, Spearman correlation = -0.42, p = 0.02). Moreover we observed an inverse association between telomere length and the presence of at least one amplicon (median length in amplified samples of 6.3 compared to unamplified, 7.4), but the comparison was underpowered and statistically not significant (Wilcoxon rank sum test, p = 0.25). These observations suggest a role for telomere attrition in promoting amplification in breast tumors.

Next, we investigated whether expression levels of genes that play a role in maintenance or manipulation of the genome varied among tumors with greater or lesser numbers of copy number aberrations. To carry out this analysis we used a second independent set of 101 ductal invasive breast tumors for which copy number profiles and Affymetrix High Throughput Array (HTA) GeneChip^® ^expression data were available (Chin *et al*., submitted). We determined the number and type of copy number changes in each tumor by counting three types of copy number alterations; copy number changes involving whole chromosomes, low level gains and losses affecting extended portions of chromosomes, and amplifications defined as focal regions of increased copy number [14]. Specifically, a clone was declared amplified if it belonged to a copy number segment <20 Mb and the increase in ratio exceeded the criterion described in the Statitical Methods. The distinction between gains and amplifications can be seen in the copy number profiles in Figure 5A. A copy number gain spanning 8q can be seen in the top left profile, while the wide variety in amplicon profiles is evident by comparison of all the profiles. We enumerate low level changes by counting "copy number transitions," the number of changes in the CGH profile from one copy number level to another that occur within chromosomes (see Supplementary Methods for further discussion of aberration finding, Additional file 2). Since the spacing between clones is ~1.5 Mb, focal aberrations that fall between clones on the array will be missed. On the other hand, all copy number transitions will be recorded, but the precision with which they will be located on the genome will depend on clone spacing. We note that these copy number analyses found that the number of copy number transitions associated with amplifications varied over a wide range in tumors of all subtypes in both datasets, however the greatest number of amplifications did not occur in the samples with either the smallest or largest number of copy number transitions (Figure 5B).

**Figure 7:**
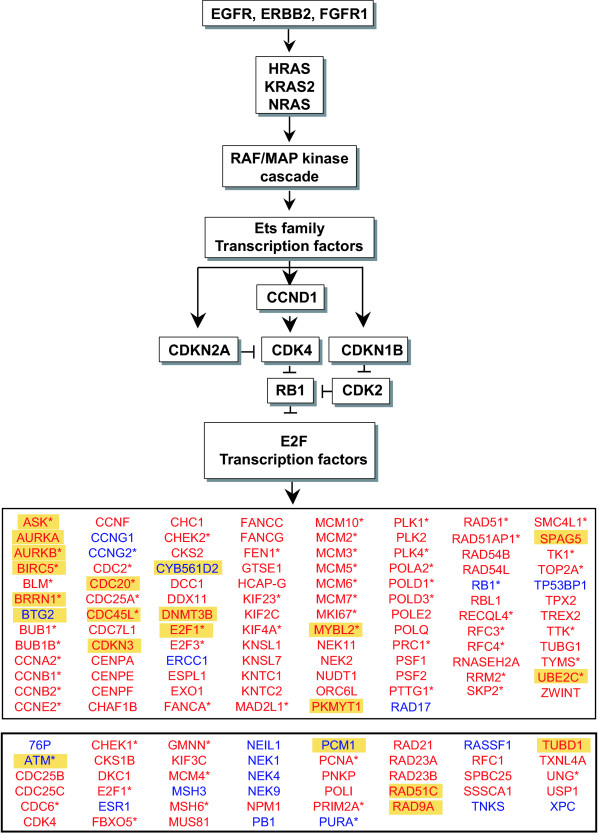
**Signaling pathway and E2F responsive genes associated with numbers of copy number transitions and amplifications**. The receptor signaling cascade impinging on Rb includes a number of up-stream genes that are frequently altered in cancers and result in deregulation of the E2F family of transcription factors through inhibition or loss of Rb function. Here, we show genes enriched for association with numbers of copy number transitions (FDR < 0.05). Genes, which have been identified as E2F targets (asterisk) and/or are correlated with *E2F1 *expression levels (|Pearson correlation| > 0.3) are shown in the top box. Genes which showed less correlation with *E2F1 *are shown in the bottom box. Genes also associated with numbers of amplifications (FDR < 0.05) are highlighted in yellow. Four additional genes associated with amplifications (FDR < 0.05) are *ASCIZ*, *MSH5*, *RAE1 *and *TDG*. Red, increased expression. Blue, decreased expression.

We tested for associations between gene expression and copy number aberrations by developing a list of 426 genes assigned to functional categories, "DNA replication," "DNA damage/repair," "cell cycle," "mitosis," "centrosome" (centrosome and centrosome cycle) and "telomere" using Gene Ontology Annotation (GOA) terms and reference to the literature (Supplementary Methods and Supplementary Table 3, Additional files 2 and 4). Many of the genes were assigned to more than one process. Expression of 350 of the 426 stability genes could be analyzed in the breast tumor data set. Controlling for estrogen receptor status as a possible confounder, we observed that the telomere functional class was significantly associated with the presence of at least one amplicon, with half of the genes showing positive and half showing negative association (Table 3). On the other hand, we found that mitosis, cell cycle, DNA replication and DNA damage/repair functional classes were highly significantly enriched for association with copy number transitions (Table 3). Moreover these associations held when only tumors of the complex subtype were considered, indicating that the results are not due to confounding between the large number of low level transitions and complex subtype. Expression of 146 stability genes was significantly associated with the number of copy number transitions (false discovery rate, FDR < 0.05). Most associations were positive (120/146 genes), indicating that enhanced expression of these genes was associated with greater numbers of copy number transitions (Figure 7). The number of amplifications was associated with mitosis, cell cycle and to a lesser extent, DNA replication categories. Again, this relation held when considering all samples, as well as only samples within the mixed amplifier subtype. Twenty-five individual genes were associated with number of amplifications (FDR < 0.05) and 21 were in common with the group of genes associated with copy number transitions (Figure 7). Here too, we observed that most associations were positive (21/25 genes), indicating that increased expression of the genes was observed in tumors with more amplifications. Finally, we investigated how the GOA categories represented by our list of 426 stability genes ranked among all known GOA categories with respect to associations with frequency of copy number aberrations by considering all probes measured by the expression analysis. In this subsequent unsupervised analysis, we found enrichment for genes associated with copy number transitions (FDR < 0.05) in the same functional classes, e.g. mitosis, cell cycle, cell division and DNA replication (Holm adjusted p-value < 0.005), providing further support for these associations.

**Table 3:** Association of expression of functional classes with copy number aberration types

**Class**	**Copy Number Transitions**	**Amplifications**	**At least one Amplicon**
Mitosis	0	4.6 × 10^-4^	0.15
Cell Cycle	0	5.2 × 10^-5^	0.02
DNA Replication	3.1 × 10^-12^	0.04	0.25
Centrosome	0.28	0.52	0.25
Telomere	0.02	0.06	2.9 × 10^-4^
DNA Damage/Repair	7.3 × 10^-9^	0.06	0.2

We noted that the 146 stability genes associated with numbers of copy number transitions included *E2F1*, and they are significantly enriched for genes known to be targets of *E2F1 *(p < 2 × 10^-6^, Fisher exact test, Figure 7). Moreover the expression levels of known *E2F1 *target genes were highly correlated with *E2F1 *expression (p < 2 × 10^-10^, Supplementary Table 4, Additional file 5). These observations provide *in vivo *validation of the *in vitro *determinations of *E2F1 *target genes. They are also consistent with deregulation of E2F being a major contributor to genomic instability affecting numbers of copy number transitions and amplifications. Taken together these observations suggest that telomere attrition and deregulated expression of genes in the other functional classes, particularly those that are targets of E2F, contribute to the numbers of chromosomal alterations.

## Discussion

Our analysis of large numbers of breast tumors by array CGH revealed variety in the numbers and types of copy number alterations in the tumor genomes. In the ductal invasive breast tumors reported here, three subtypes were distinguished by copy number alterations. The subtypes differed with respect to the numbers and types of aberrations, as well as patient survival. The1q/16q subtype with very few copy number alterations in addition to gain of 1q and loss of 16q was associated with the best patient outcome, consistent with other studies. Searches for tumor suppressor gene(s) on 16q have failed to find mutations in candidate genes in the region in ductal invasive breast cancer, although mutations in E cadherin and loss of 16q are characteristic of lobular breast tumors. Two genes involved in telomere maintenance, *TERF2 *and *TERF2IP *were among those ruled out as tumor suppressors on 16q, as was *E2F4 *[15-17]. The stability of the genome of these tumors also suggests that copy number alterations of these and other stability genes mapping within the aberrant regions, +1q and -16q are less likely to contribute to chromosomal level instability in breast cancer.

Complex tumors with extensive chromosomal level instability were associated with poor patient survival. They are similar to *BRCA1 *hereditary tumors in their copy number alterations [18,19] (Figure 3). *BRCA1 *participates in a number of cell functions that maintain genome integrity either directly through double strand break repair or indirectly through maintenance of checkpoints at G1, S and mitosis [20-22]. Thus, it is possible that *BRCA1 *[23,24] or the genes/pathways that interact with *BRCA1 *are defective in this subtype either through mutation, silencing or copy number mediated dosage effects. We note that the copy number loss on 17q associated with this subtype includes the *BRCA1 *locus (9/16 tumors, Figure 4).

The discrimination of breast tumor subtypes based on copy number aberrations led us to investigate possible associations of copy number aberration types with alterations in processes/genes involved in maintenance of genome stability. We observed shorter telomeres in tumors with greater numbers of amplifications, consistent with telomere attrition promoting this type of copy number aberration in breast tumors. Telomere dysfunction, often referred to as "telomere crisis" has been implicated in amplification, particularly by breakage-fusion-bridge processes. On the other hand, our analyses of stability gene expression in relation to copy number aberration types found that expression of genes in the functional classes; "mitosis," "cell cycle," "replication," and "DNA damage/repair" were associated with greater numbers of copy number transitions. Furthermore, a subsequent analysis found significant enrichment for these same classes among all GOA groups when analyzed with GOStats [25]. The number of amplicons was associated with similar functional groups, "mitosis" and "cell cycle." Many of these genes are E2F targets [26-36] and therefore potentially coordinately deregulated due to Rb pathway defects [37]. Abrogation of Rb pathway function is frequent in breast tumors by loss of expression of Rb or altered expression of inhibitors of Rb activity (e.g. loss/silencing of *CDKN2A *(p16) and amplification and/or over expression of *CCND1*, *CDK4*, *CDK6*) (Figure 7). It is interesting to note that whereas *E2F1 *is up-regulated in breast tumors, its expression is low in prostate tumors [38], which typically have genomes with fewer copy number changes than most ductal invasive breast cancers [39]. For example, in an array CGH dataset of 64 primary prostate tumor samples [39], the median number of copy number transitions was 13 per tumor genome compared to 30 in our primary breast tumor samples (p < 5 × 10^-9^, Wilcoxon rank sum test). Mechanistic support for a central role of *E2F1 *in genomic instability comes from a recent report that elevated numbers of DNA double strand breaks are present in cell lines with deregulated *E2F1 *and Rb deficiency [40].

Chromosomal instability has been observed *in vitro *when many of these E2F target genes (Figure 7) associated with replication, DNA repair, cell cycle control and the mitotic checkpoint are mutated, knocked out or knocked down using siRNA [8,41,42]. Contrary to expectation, we observed that greater chromosomal instability in breast tumors is associated with increased expression levels of many of these genes, even though they have loss of function instability phenotypes. These assays further demonstrate that loss of a single copy of some of the genes results instability or cancer prone phenotypes. Genes that have been shown to be haploinsufficient in this way and that are among those we identified as showing significant association with the number of copy number aberrations in our tumors (FDR < 0.05) include *RAD17*, *ATM *and *RB1*, which are expressed at lower levels in tumors with more copy number changes. These genes are also negatively correlated with *E2F1 *expression. Other genes showing haploinsufficiency *in vitro*, *MAD2L1*, *PLK4*, *BUB1B *and *CHEK1 *show enhanced expression in association with number of chromosomal changes and are positively correlated with *E2F1 *expression (Supplementary Table 4, Additional file 5). As all seven of the above mentioned genes with haploinsufficiency phenotypes map to regions of frequent loss in breast tumors and genetic instability phenotypes are associated with deficiency in these genes, we asked whether loss of function might play a role in the subset of tumors in which there is a copy number loss of the locus. Specifically, we asked if their expression levels were down regulated when there is a copy number loss. Although 118 of the genome stability genes showed highly significant reduction in expression in tumors in which the locus was lost (FDR < 0.05, one-sided Wilcoxon rank sum test), we found little difference in expression level with copy number loss for *MAD2L1*, *PLK4*, *ATM *and *RB1*, whereas *BUB1B *was increased in expression in tumors with loss of the locus (Supplementary Table 4, Additional file 5). Only expression of *RAD17 *was significantly reduced when lost (unadjusted p = 8 × 10^-4^, Wilcoxon rank sum test), suggesting that *RAD17 *might be haploinsufficient in tumors with copy number loss of the locus at 5q13.

Our observations in tumors support the hypothesis that global alteration of expression of genes involved in processes such as chromosome segregation and maintenance of genome integrity, driven by deregulation of E2F, underlies much of the chromosomal instability in breast tumors. Furthermore gene expression appears to be relatively up-regulated. On the one hand, this observation seems contradictory in light of the phenotypes resulting from mutational analyses of genes involved in maintenance of genome stability. Such *in vitro *studies have generally assessed the consequences of functional deficiency one gene at a time and have found that individually many genes have loss of function instability phenotypes. On the other hand, as many of these genes participate in multi-protein complexes that depend on proper stoichiometry for function, alterations resulting in overproduction or deficiency are likely to have similar or related phenotypes (reviewed in [43]). Indeed, in mammalian cells, instability phenotypes have been reported in association with both up and down regulation of genes such as *MAD2L1 *[8,41], *ATR *[44,45], *PLK4 *[46] and *AURKA *[47]. Further studies will be required not only to assess instability phenotypes when expression levels are increased, but also how phenotypes might vary when multiple genes are up-regulated.

In tumors, changes in gene dosage due to low level copy number alterations may also lead to small alterations in expression of multiple genes, which together could contribute to dysfunction of processes manipulating the genome, resulting in more error prone cell division cycles. Thus, during tumor progression, genome instability may be enhanced not only by deregulation of E2F, but also by the acquisition of greater numbers of copy number changes encompassing more genes involved in genome maintenance. Since genetic instability is an on-going feature of tumors, allowing them to evolve resistance to therapy, the ability to recognize the active mechanisms of instability in tumors may help to guide therapeutic decisions.

## Conclusion

Application of array CGH to the study of breast tumors found three subtypes. Investigation of the numbers and types of copy number alterations in tumors and their association with differential expression of genes likely to play a role in manipulation or maintenance of the genome implicated telomere dysfunction in the propensity to amplify. On the other hand, the number of lower magnitude gains and losses of chromosomal segments was associated with differential expression of genes which were significantly enriched for the known targets of E2F, supporting the hypothesis that deregulation of E2F underlies much of the chromosomal instability in breast tumors. Furthermore, we observed enhanced expression of most E2F target genes, indicating that over expression rather than deficiency was associated with genetic instability. These observations provide a possible explanation for the failure to find mutations in sporadic tumors in genes that have roles in maintenance or manipulation of the genome.

## Competing interests

The author(s) declare that they have no competing interests.

## Authors' contributions

JF performed the statistical analysis and contributed to writing the manuscript, AMS and BY performed the array CGH and contributed to writing the manuscript, HL performed the telomere length measurements, AO performed statistical analysis, RS performed array CGH, SD directed the dissection of tumor tissue and extraction of nucleic acids, TT contributed to the development of analytical procedures for image analysis and performed the image analysis, BML was responsible for pathologic diagnosis, AJN contributed to the development of analytical procedures, JM and JZ manage and direct the UCSF Comprehensive Cancer Center Risk Program and provided access to *BRCA1 *mutant tumors, KC and SD performed the array CGH for breast study II, HF was responsible for the expression array measurements, JG and FW directed breast study II and shared their unpublished data, DP participated in technology development that made this study possible, DGA directed the study, participated in technology development and was responsible for preparation of the manuscript.

## Pre-publication history

The pre-publication history for this paper can be accessed here:



## Supplementary Material

Additional File 1 **Additional file 1 – Supplementary Table 1. Patient Characteristics**Provides information on patient samples. Click here for file

Additional File 2 **Additional file 2 – Supplementary Statistical Methods **Provides a detailed description of statistical methods. Click here for file

Additional File 3 **Additional file 3 – Supplementary Table 2. Array CGH data **Provides array CGH log_2_ratios for clones and genome order for all samples. Click here for file

Additional File 4 **Additional file 4 – Supplementary Table 3. Genome stability genes and associations with copy number aberrations **Provides information on 426 genome stability genes and associations with copy number aberration types. Click here for file

Additional File 5 **Additional file 5 – Supplementary Table 4. Genome stability genes and their relation to E2F. **Table provides information on whether the genes are known targets of *E2F1*, correlation of their expression with *E2F1*, correlation of their expression with copy number in samples in which copy number of the locus is reduced. Click here for file
