# Supplementary material for: Breast tumor copy number aberration phenotypes and genomic instability
Source: BMC Cancer. 2006 Apr 18;6:96. doi: 10.1186/1471-2407-6-96 (PMC1459181; doi:10.1186/1471-2407-6-96)
Supplement: Additional File 2 — – Supplementary Statistical Methods Provides a detailed description of statistical methods. [file 1471-2407-6-96-S2.doc]

**Supplementary Statistical Methods**

**Array data processing.** We used the custom program, SPROC to automatically filter the data to reject data points based on low DAPI intensity, low correlation between Cy3 and Cy5 within each segmented spot and low reference/DAPI signal intensity. Clone ratios were declared as missing if ratios were missing more than two of the three replicate spots for each clone or the standard deviation of the replicates was greater than 0.2. Since the samples in the dataset were hybridized to two different versions of the genome scanning array (HumArray1.14 and HumArray2.0), the data were combined by considering only the clones present on all arrays. Before combining data, clones were removed if they were not mapped on the July 2003 freeze of the human genome sequence, were present in fewer than 50% of the samples on either array version or were known to contain common copy number variants. Duplicate clones were averaged. The final dataset contained 2044 unique BACs, and clone values were missing in a median of 7.5% of the samples. For each tumor, we plotted the data in genome order as the mean log_2_ratio of the replicate spots for each clone normalized to the genome median log_2_ratio. These data are available in Supplementary Table 2.

Affymetrix HGU133A data were obtained from Chin *et al*. (submitted). For data processing multi-chip robust normalization was performed using RMA software [1]. Transcripts assessed on the arrays were classified into two groups by considering the joint distribution of the median and standard deviation of each probe set across samples. To classify transcripts, we used Gaussian model-based clustering. For computational feasibility, 2000 probe intensities were randomly sampled and clustered using *mclust* [2, 3] with two clusters and unequal variance. Next, the remaining probe intensities were classified into the newly created clusters using linear discriminant analysis. As a result, genes were allocated into two groups according to whether both variability and median of their intensities across the samples were small (group 1) or either median or variability were larger (group 2). The cluster containing probe intensities with smaller mean and variance was excluded from the analysis and the second cluster was considered “measured” for the purposes of this study.

**Identification of copy number changes**. The array CGH data were analyzed using circular binary segmentation (CBS) [4] with default parameters to translate experimental intensity measurements into regions of equal copy number as implemented in the DNA copy R/Bioconductor package. Missing values for clones mapping within segmented regions of equal copy number were imputed by using the value of the corresponding segment. A few clones with missing values (< 0.5%) were located between segmented regions and their values were imputed using the maximum value of the two flanking segments. Thus, each clone was assigned a segment value referred to as its “smoothed” value. The scaled median absolute deviation (MAD) of the difference between the observed and smoothed values was used to estimate the tumor-specific experimental variation. All of the tumors had MAD less than 0.25. The gain and loss status for each probe was assigned using the mergeLevel procedure [5]. In this process, segmental values across the genome were merged to create a common set of copy number levels for each individual tumor. The minimum and maximum criteria used in mergeLevels were 0.05 and 0.25, respectively. The clones corresponding to the copy number level with the smallest absolute median value were declared unchanged whereas all the other clones were either gained or lost depending on the sign of the segment mean.

The frequency of alterations at each clone was computed as the proportion of samples showing an alteration at that locus. The extent of the genome assigned to each clone was computed by assigning a genomic distance equal to half the distance to the two neighboring clones or to the end of a chromosome for clones with only one neighbor. The number of copy number transitions was computed based on the initial CBS segmentation by counting the number of segments in the genome minus the number of chromosomes. Finally, to identify single technical or biological outliers such as high level amplifications, the presence of the outliers within a segment was allowed by assigning the original observed log2ratio to the clones for which the observed values were more than four tumor-specific MAD away from the smoothed values. The amplification status for a clone was then determined by considering the width of the segment to which that clone belonged (0, if an outlier) and a minimum difference between the smoothed value of the clone (observed value, if an outlier) and the segment means of the neighboring segments. The clone was declared amplified if it belonged to the segment spanning less than 20 Mb and the minimum difference was greater than exp(-x^3^) where x is the final smoothed value for the clone. Note that this procedure allowed clones with small log2ratio to be declared amplified if they were high relative to the surrounding clones with the required difference becoming larger as the value of the clone gets smaller (e.g. a difference of 1 is required when the clone value is 0 and 0.36 when the clone value is 1). This approach was motivated by the observation that high level amplifications may arise within regions of copy number loss and thus it is necessary to consider the relative magnitude of the aberration, rather than simply absolute magnitude. Regions of high level amplification were declared to be recurrent when present in at least two samples. The clones were further manually grouped to form contiguous regions thereby referred to as amplicons. Each sample was further classified as amplified for a given amplicon if it contained at least one amplified probe in the amplicon region. Minimal regions of recurrent amplification were determined by manual inspection of copy number profiles.

The number of partial chromosome arm and centromeric copy number transitions, whole chromosome changes, and number of chromosomal arms with an amplification were calculated as follows. Total number of copy number transitions was defined as the total number of segments minus the number of chromosomes. Centromeric copy number transitions were defined as occurring when the segment end was assigned at the most proximal clone on the p-arm. Whole chromosome changes were assigned to chromosomes without identified breakpoints and when the chromosomal segment mapped to the gain or loss level. Finally, an autosomal chromosome arm was scored as amplified if it contained at least one amplified clone. We assigned “amplifier status” to each sample that contained at least one amplification event.

**Clustering tumor profiles***.*  Tumor profiles were clustered using smoothed imputed data with outliers present. Agglomerative hierarchical clustering with Pearson correlation as a similarity measure and the Ward method to minimize sum of variances were used to produce compact spherical clusters [6]. The number of groups was assessed qualitatively by considering the shape of the clustering dendogram.

**Within subtype similarity of tumor profiles.**

One way to assess this similarity is the median pairwise Pearson correlation, which for hereditary *BRCA1* mutant tumors is 41%. In the complex tumors it is 33% compared to 76% in the 1q/16q tumors and 34% in the more heterogeneous third group of mixed amplifier tumors. A second measure of intra-class similarity, which reflects the extent of recurrence of the copy changes, is to ask what proportion of autosomal clones that are altered in at least 20% of the cases are also altered in 50%. In hereditary *BRCA1* mutant tumors, this proportion is 32%; it is 47% in the complex tumor subtype, 34% in the 1q/16q group (reduced because of the gain of chromosome 7, which is present in some cases) and only 21% in the third group of mixed amplifier tumors. Note that the estimate for hereditary *BRAC1* tumors is unreliable because of the small (5) number of tumors. Overall, by this second measure, complex tumors display the highest degree of within group similarity in spite of a high frequency of copy number alterations.

**Association of copy number with phenotypes***.*  Smoothed imputed data with outliers present was used in association studies with the phenotypes such as estrogen receptor or *TP53* mutation status. The use of smoothed, rather than observed data has been previously shown to dramatically increase sensitivity without sacrificing specificity of the tests [5]. The standardized linear regression coefficient was used as a test statistic. The corresponding raw p-values were adjusted using the False Discovery Rate [7]. Significance was claimed at the FDR < 0.05, which corresponds to the expectation of at most 5% of false discoveries among the loci declared significant.

**Association of expression of genes involved in manipulation or maintenance of the genome with the copy number aberrations.** The seven functional groups implicated in the instability process were defined by querying for GOA terms “centrosome,” “centrosome cycle,” “mitosis,” “repair,” “damage,” “cell cycle” and “telomere” using the R/Bioconductor package biomaRt [8]. The resulting list of genes was then cross-referenced with a manually developed list of genes known to play a role in manipulation or maintenance of the genome. The same genes could be assigned several functions. The biomaRt package was employed once again to assign *Entrez* and RefSeq IDs to the genes. We then used HGU133A annotation R-meta package to identify Affymetrix probes corresponding to each gene by matching based on Entrez Gene IDs. Note that only probes that were classified as “measured” were used in the further analysis. We observed that the genome stability genes were highly enriched for probes considered “measured,” as they had higher or more variable expression levels (see Methods); 89% of the probes corresponding to these genes had at least one “measured” probe as opposed to 61% of genes overall. We mapped the genes onto the genome and found no evidence of spatial co-clustering.

We considered three types of genomic instabilities: total number of copy number transitions, number of amplified chromosome arms and binarized amplification status of each sample (i.e. presence of at least one amplification in a sample). The standardized regression coefficient was employed as a test statistic for each of the three types of instability. To account for a likely source of variation and possible confounding, all linear regressions were controlled for ER status by explicitly including the ER variable as a covariate into the regression. For each gene with at least one expressed probe, we calculated the test statistic for each probe as well as the corresponding p-values for the standardized regression coefficient. The p-values for association of individual probes were adjusted using FDR and the significance was declared at FDR < 0.05. To assess significance of the individual functional groups, we used the Wilcoxon rank-sum test to assess the difference in the strength of association between the expressed probes corresponding to the genes in that group versus all the other expressed probes on the array. The group was declared to be significantly enriched for association with a particular type of instability if the corresponding Wilcoxon rank sum test p-value was less than 0.05. No p-value adjustment was done here due to the limited number (6) of tests performed for each type of instability.

**Assignment of copy number to each gene and association of copy number with expression***.*  Each gene was assigned an observed copy number and alteration (gain/loss) status of the nearest mapped BAC on the array. The majority of genes (76%) had a nearest clone within 1 Mb and 60% had a clone within 500 kb. Using the data set from Chin et al. (submitted), we calculated Spearman correlations between expression level and copy number for each stability gene. In the case of multiple probes, the maximum correlation over the probes was shown.

**Determination of association of gene expression with E2F1 expression or copy number loss**. Each gene was associated with the expression of the probe that gave the highest correlation with the gene’s copy number. The Pearson correlation was computed with *E2F1* expression and expression of the remaining genes. Genes with Pearson correlation greater than 0.3 were considered to be associated with *E2F1*. Enrichment of the correlated genes for known *E2F1* targets was determined using the Wilcoxon rank sum test treating correlations with *E2F1* as continuous values, as well as the Fisher exact test with correlations with *E2F1* binarized at a threshold of 0.3. As expected known *E2F1* targets were significantly enriched for the genes whose expression was highly correlated with *E2F1* (p << 0.001: Wilcoxon rank sum test and Fisher exact test).

One-sided Wilcoxon rank sum test was employed to compute the significance of reduced expression when copy number loss occurred relative to when the copy number of the locus was unaltered. The resulting p-values were adjusted using FDR. Note that samples with a copy number gain of the locus were excluded from the comparison.

**References**

1. Irizarry R, Bolstad B, Collin F, Cope L, Hobbs B, Speed T: **Summaries of Affymetrix GeneChip probe level data**. *Nucleic Acids Research* 2003, **31**:e15.

2. Yeung KY, Fraley C, Murua A, Raftery AE, Ruzzo WL: **Model-based clustering and data transformations for gene expression data**. *Bioinformatics* 2001, **17**(10):977-987.

3. Yeung KY, Medvedovic M, Bumgarner RE: **From co-expression to co-regulation: how many microarray experiments do we need?** *Genome Biol* 2004, **5**(7):R48.

4. Olshen AB, Venkatraman ES, Lucito R, Wigler M: **Circular binary segmentation for the analysis of array-based DNA copy number data**. *Biostatistics* 2004, **5**(4):557-572.

5. Willenbrock H, Fridlyand J: **A comparison study: applying segmentation to array CGH data for downstream analyses**. *Bioinformatics* 2005, **21**(22):4084-4091.

6. Hartigan JA: **Clustering Algorithms**. New York: Wiley; 1975.

7. Hochberg YBY: **Controlling the False Discovery Rate: A Practical and Powerful Approach to Multiple Testing**. *Journal of the Royal Statistical Society Series B* 1995, **57**(1):289-300.

8. Durinck S, Moreau Y, Kasprzyk A, Davis S, De Moor B, Brazma A, Huber W: **BioMart and Bioconductor: a powerful link between biological databases and microarray data analysis**. *Bioinformatics* 2005, **21**(16):3439-3440.
